# Crossing Social Boundaries in an Immigration Context: Exogamy and Gendered Employment Patterns in Unions in Germany

**DOI:** 10.1007/s12147-021-09281-8

**Published:** 2021-06-10

**Authors:** Mirko K. Braack, Nadja Milewski, Heike Trappe

**Affiliations:** Institute for Sociology and Demography, University of Rostock, Rostock, Germany

**Keywords:** Gendered employment patterns, Exogamous unions, Gender equality

## Abstract

We study gendered employment patterns in unions by focusing on the role of exogamy for non-migrants in Germany. Classical assimilation theory has studied such mixed migrant-non-migrant unions mainly with a focus on the members of ethnic minorities. However, this perspective neglects the question of the social consequences of exogamy for the members of the majority group. We aim to fill this knowledge gap by investigating the association of being in a mixed union and the employment patterns of the couple. Our theoretical considerations and working hypotheses are derived from modernization theories, welfare state and labor market theories, gender studies, and social boundary-crossing frameworks. Drawing on the scientific use file of the German Microcensus of 2013, our sample consists of 44,499 non-migrant men (about 7% of whom are in a mixed union with a migrant) and 43,722 non-migrant women (about 5% of whom are in a mixed union). We estimate multinomial logistic regression models. We conclude that the persistent disadvantage for immigrants on the labor market in Germany shapes the gendered employment patterns of their unions, which, in turn, affect the members of the majority population. For non-migrant men, exogamy is associated with a re-traditionalization of employment patterns, whereby a man is more likely to be the main earner if he is in an exogamous union than if he is in an endogamous union. For non-migrant women, by contrast, we find evidence of a role reversal in exogamous unions, whereby the woman is more likely to be the main earner.

## Introduction: Gender Roles, Migration, and Mixed Unions

Many researchers and politicians perceive gender equality as one—if not *the*—dividing line between the post-industrial countries on the one hand, and the industrial and agrarian countries on the other [51]. Likewise, as levels of immigration and of religious diversity increase in Europe, gender equality has become a controversial topic *within* European countries with respect to the migrants’ participation in the host society. By dichotomizing migrants and majority populations into a “traditional/gender-inequality” group and a “modern/ gender-equal” group, issues of gender equality reproduce and contribute to stereotypes, xenophobia, and femonationalism by ignoring the heterogeneity within mainstream populations, and stigmatizing non-European migrants [29]. Previous research on gender-role behavior has mainly investigated the gendered employment patterns of the majority populations and of immigrants, or, more generally, of ethnic minorities separately. Our study focuses on a group of people who have crossed the social boundaries between immigrant minorities and members of the majority group in the most intimate of life domains: i.e., exogamous couples [7]. Mixed unions between an immigrant and a non-migrant provide a “microlaboratory of intercultural relations” [90: p. 25] for studying the scope, the internal dynamics of conflict and negotiation, and the social consequences of such boundary crossing. The aim of our study is to compare the gendered employment patterns of exogamous unions with the patterns of non-migrant endogamous unions in Germany. In so doing, we are interested in the question what role exogamy plays for the majority population.

Due to selection into mixed migrant-non-migrant unions, the characteristics of the partners in exogamous unions differ from those of the partners in endogamous unions in terms of their socio-demographic determinants and attitudes [10, 26, 39, 75]. The migrant partners are subject to particular legal conditions and have migration-specific characteristics that have long-lasting effects on their post-migration life course. We argue that the structural conditions of the migrant partner, which may include having less host country-specific cultural capital than the non-migrant partner, may also affect how the couples organize their life together, and, in turn, the life of the non-migrant partner. Thus, these conditions may also cause the arrangements of non-migrants in exogamous unions to differ from those of non-migrants in endogamous unions. Our research question is as follows: *How are couples’ gendered employment patterns associated with exogamy, i.e., having a migrant partner?* In addition, to investigate this question in more detail, we pay attention to differences in legal conditions of migrants and ask: *Does the regional context of the origin of the migrant partner affect the couple’s employment patterns*?

Our theoretical considerations and working hypotheses are derived from modernization theories, welfare state and labor market theories, gender studies, and social boundary-crossing frameworks, which are accompanied by issues related to international migration and the presence of migrants in the labor and partner markets [7, 41, 57]. The gender-based division of labor in the family is one of the main indicators of gender inequality, because the security, quality, and status of employment differ for men and women [28: pp. 23–46]. Like elsewhere in the Global North, the structural conditions for women in Germany have changed in recent decades [78: p. 87]. In addition, welfare states and the employment patterns of men and women have evolved over time [100]. Among the consequences of these changes are that increasing shares of women are participating in the labor market [20], family lifestyles are pluralizing [44], and women are becoming less dependent on men [27]. Nauck had pointed out in the beginning of the 1990s that this kind of research has mainly focused on the German mainstream population [79: pp. 704 f.]; i.e., on non-migrants. Up to today, studies on “mainstream “ family dynamics in Germany have failed to recognize that the presence of immigrants may affect the German population as a whole on the macro level. Likewise, mainstream research has ignored the question of whether the life courses of non-migrant individuals may be affected by immigration, or, more specifically, by the crossing of social boundaries between majority and minority groups, which is the case in mixed marriages involving an immigrant [2].

Previous research that has addressed the topic of exogamy has focused almost exclusively on immigrants. The most common way to describe migrants and their contacts with non-migrants is in terms of “integration” and “assimilation” theories. This approach posits that being in a mixed union is both a means and a result of adaptation processes; and, thus, that exogamy indicates that members of a minority group have become part of the host society or the majority population [3, 40]. Obviously, this approach is often based on a deficit-oriented perspective on immigrants. It is connected to “an imagined project of ‘us’” [94: p. 1] in reference to the majority population. The research on mixed unions conducted in Germany and elsewhere has mainly concentrated on the migrant partner [39] and has rarely looked at the non-migrant partner. Of the few studies that took the non-migrant partner into account, most had qualitative research designs [36]. However, in some recent quantitative studies on European countries, the theoretical link between migrant assimilation and mixed unions has been challenged [10, 75, 97]. Power imbalances between the non-migrant and the immigrant partners have been recognized. For example, the migrant partner may have less power because s/he faces restrictions associated with his/her legal status, is subject to discrimination, or has lower earnings potential than non-migrants [1, 6, 66]. Our aim is to contribute to this new strand of empirical research on mixed unions from the perspective of the majority population by focusing on gendered employment patterns in a quantitative study based on large-scale representative data, and by comparing them to endogamous non-migrant unions in Germany.

## Employment Patterns, Gender Roles, and Institutional Influence

In this section, we provide a short review of the gendered employment patterns in Germany, and of how these patterns differ in eastern and western Germany. Furthermore, we summarize research on mixed unions, and what is known about the gender roles in these partnerships. We conclude this section by formulating our working hypotheses.

Although it is likely that a “pure male breadwinner model never existed” [67: p. 153], this model has long been a societal ideal for western countries: i.e., the husband earns the family income, and the wife is supported by this income while assuming responsibility for the care work in the family. Modernization theories highlight that the more women achieve higher educational attainment and participate in the labor market, the more they are able to lead an independent life. This evolution is associated with a pluralization of lifestyles and for women with an increasing orientation toward activities outside of the household [87]. Changes in welfare state policies and gender relations have accompanied this more gender-neutral point of view [70], and have thus provided a wider range of employment patterns.

### The German Context

In contrast to other European countries, Germany is not (or is not yet) dominated by a single new family model, but instead has a number of different gendered employment patterns: i.e., male breadwinner, one-and-a-half-earner (with the man in full-time and the woman in part-time employment), dual-earner, and female breadwinner arrangements [20, 24, 67]. Which of these models people follow can vary over the life course depending on whether they have children, and, if so, how many children are living in the household, and how old the children are. The partners must negotiate their arrangements to get the “ideal” outcome for both of them. On the one hand, the positions on the labor market and the preferences of the partners affect their labor market participation. On the other hand, the welfare state provides incentives for a gendered division of labor [70]. On the macro level, gendered patterns of child care responsibilities and labor market participation are the result of different family and employment policies. Among the policies that both influence and are influenced by the gendered division of labor are working time regulations, family leave policies, and the availability of early child care [41]. Cross-national comparisons suggest that different gender arrangements are shaped by and embedded in cultures, which construct “the idea of the ‘ideal’ family form” [84: p. 533].

Like in other countries, one aim of family policies in Germany is to facilitate the compatibility of care work, including child care, and paid labor [12]. The welfare state policy approaches used to achieve this goal differ across the Nordic social democratic countries, the conservative Central European countries like Germany, and the liberal welfare state countries like the U.S. The Nordic countries support dual-earner and egalitarian arrangements by promoting women’s participation in both paid and family care work. Meanwhile, the conservative countries also provide support for women to engage in family care work, but they do not promote gender equality in the division of labor, including the division of paid working hours [41, 68].

However, Germany’s current policy approach seems ambivalent. Different measures support different models simultaneously, which might relate to the country’s history of political division. Germany represents a special case due to the diversity of cultural traditions *within* the country. The gendered division of labor evolved in different ways in eastern and western Germany as a consequence of their different political systems between 1949 and 1989. Thus, prior to reunification in 1990, eastern and western Germany had different gendered policies and outcomes [91: p. 108]. In short, because of these different policy approaches, the former GDR (eastern Germany) encouraged more gender-egalitarian arrangements, whereas the former FRG (western Germany) generally supported the male breadwinner model [18, 100]. Since 1990, a trend toward convergence in the two parts of the country has been observed. This convergence was caused by the increased adoption of the one-and-a-half-earner model across Germany, which led the two parts of the country to adjust their labor market participation levels accordingly [91]. There are, however, still differences in the employment patterns of women in eastern and western Germany that are based in part on the prevailing motherhood ideals [85]. In western Germany, like in other western countries, mothers tend to reduce or end their participation in paid work after having children [19]. In eastern Germany, by contrast, mothers are less likely to leave the labor market, or to reduce their working hours [99]. Moreover, in recent years, the female breadwinner arrangement has become increasingly common, especially in eastern Germany [53: p. 1734]. However, this arrangement cannot be described as a male breadwinner arrangement with changed gender roles, as it appears to be a rather fragile arrangement that can be challenging for both partners [62], in part because women are more likely than male breadwinners to work in atypical or low-skilled employment [78: p. 164]. A very recent study on female breadwinner couples has shown that female breadwinning is associated with a higher risk of having a low household income, as women tend to earn less than men [64].

Changes in gendered employment patterns are not just the result of policy changes, but a long-term, three-way process [88: pp. 77–85] consisting of post-industrialization, which is connected to a rising number of women in paid employment; expansion in education; and a liberalization process that has led to shifts in values [51]. In this paper, we want to draw attention to a fourth process: i.e., migration and increasing population diversity [4]. Up to now, this process has rarely been considered in the research on gendered employment patterns. We argue that immigration from other European and non-European countries, in part through more globalized labor and partner markets, may also affect the members of the majority population. Of the resident population in Germany today, more than 10 million people immigrated either as adults or as children of immigrant parents, and the migrant population, including the German-born descendants of immigrants, make up one-fifth of the total population (in 2013). The main countries of origin of these immigrants are Turkey, Poland, Russia, Romania, and Italy [22]. The number of mixed unions in (western) Germany grew quickly between 1950 and 1980, and then slowed down. In recent years, the share of mixed marriages in all marriages has been relatively stable, at around 6% [47]. Haug as well as Nauck observed that the country of origin of the migrant partner tends to differ depending on their gender [47, 80]. Until 1995, women of the majority group were more likely than men to have a migrant partner; but since 1995, non-migrant men have been more likely than non-migrant women to have a migrant partner. German non-migrant women are more likely to have a partner from one of the large minority groups (migrants from Turkey or Italy) or from other former “guest worker” countries. Meanwhile, German non-migrant men are more likely to have a migrant partner from Poland, Turkey, Thailand, Brazil, or the Philippines. This pattern is in line with trends observed in other European countries; i.e., that non-migrant women are more likely in their partner search to look for a partner with cultural proximity who already lives in the country, while men are more likely to look at a wider geographical range of possibilities [46, 96].

### Diverse Partner Markets and Non-Migrants in Mixed Unions

If immigration increases, the mate selection process of non-migrants may change. This may, in turn, create new challenges for couples in negotiating employment patterns. For example, mixed couples may have to deal with legal barriers, with cultural differences in gender role behavior, and with inequalities that affect the ability of migrants to participate in the labor market. Thus, in this section, we discuss the factors that affect mixed unions, and the consequences mixed unions experience at the individual and the societal level [83].

In most European countries, the numbers and shares of mixed unions are rising [65]. In recent years, research on mixed unions has been increasing as well. The causes and the consequences of these mixed unions are generally discussed [26, 106] in two theoretical frameworks: as a status exchange [45]; or as a form of positive assortative mating, and the realization of homogamy preferences [55]. Both of these perspectives highlight the influence of education on partner choice [35]. Some studies has examined the determinants of partner choice, such as the influence of opportunities and structures (for an overview: [83, 90]). A smaller number of studies have looked at how being in a mixed marriage affects health [75], the likelihood of union dissolution [76] or of forming a union at later ages [30], and subsequent union formation [86, 102]. Most studies on exogamous unions in Germany have focused on the migrant partner [5, 31, 39, 50, 58, 92]. Only a few of these studies have taken the perspective of the non-migrant partner and his/her lived experiences and social consequences. They showed that exogamous unions could be a secondary partner market for people who had no or less access to other partner markets in Germany, like the labor market [38], and that these individuals are more likely to meet a migrant partner during leisure activities [105].

Although much of this literature has focused on attitudes toward gender equality and related behavior, to the best of our knowledge, no existing study has examined gendered employment patterns in mixed unions in the majority group by applying a couple perspective; i.e., by looking at the non-migrant and his/her partner. A recent study on non-migrants in several European countries explored individual attitudes of non-migrants in exogamous and endogamous unions toward gender equality [10], and found that non-migrants in mixed unions were more supportive of gender equality than their counterparts in endogamous unions. This finding contradicts the stereotyping that often occurs in the media [108] or in research on mixed marriages, such as studies on German men who are married to women from Asia [66] or on German women who are in romantic relationships with men from Sub-Saharan Africa [11].

As Schinkel noted, for migrants, “there is no socio-economic status high enough, no cultural assimilation perfect enough […] that qualifies them or their ‘group’ as unproblematically part of ‘society’” [93: p. 7]. Thus, there is an imbalance between the partners because their individual status in society is not the same [6, 16]. Differences in the partners’ legal status are especially salient when the mixed union involves a non-EU migrant, rather than a more privileged EU migrant, because German immigration policies have different targets and conditions for different types of migrants based on their country of origin [33]. Since 2005, citizens of the European Union have freedom of movement (and settlement), and thus have the right to move and seek employment without restrictions in all of the member states of the European Union. By contrast, to enter an EU country as a labor migrant, a non-European needs to have a vocational qualification, and has a limited time to find a job. Moreover, non-migrants and citizens of the European Union have a higher occupational status than migrants [49: pp. 25f.]. Thus, for non-migrants, it has become easier to meet and mate with partners not only from abroad (marriage migrants) or from the older guest worker immigrant population, but from new intra-European migrant groups [95, 103].

### Non-Migrants and Immigrants Participating in the Labor Market

Labor market participation is one of the crucial indicators in theoretical frameworks of immigrants’ access to different parts of the host society [82]. As such, the labor market is a place in which inequality and discrimination become visible. Labor market opportunities and barriers are different for different parts of the society. For example, women, older people, and migrants are especially likely to face barriers in employment [48]. In industrial sectors of the labor market, women are underrepresented, and may be confronted with negative stereotypes toward women in industry [81]. One-third of the unemployed population of working age in Germany are migrants or descendants of migrants [13]. In particular, migrants from non-European countries (with the exception of migrants from the U.S. and Canada) tend to be more disadvantaged than German non-migrants in terms of their socioeconomic status and their labor market participation rates [57, 74]. Furthermore, there are some gender differences among migrants. While women from non-European countries are less likely than their male counterparts to participate in the German labor market, the labor market participation levels of women from former socialist countries, which promoted more gender equality, do not differ from those of men [32].

Many of these differences between migrants and non-migrants can be attributed to discrimination by employers or by the host society [54], and to differences in the resources and the educational and vocational qualifications of non-migrants and migrants [43].

While the German state has enacted laws designed to increase the migrants’ participation in the labor market, there are gaps in these regulations. They do not take sufficiently into account the special needs of migrants, such as the accreditation of skills [98]. Moreover, there is a tight linkage between vocational education and occupation in Germany, which, together with the downgrading of migrants’ educational levels, lead to migrants having disadvantages and lower status on the labor market. While the participation levels of immigrants gradually converge with those of non-migrants with their duration of stay in the host society, immigrants consistently have lower earnings, higher unemployment rates, and longer durations of transitions to stable employment than non-migrants [9, 17]. With respect to such disadvantages, Germany is clearly no exception, as migrants face disadvantages on the labor market in many other countries as well. For example, in the U.S. and Canada, migrant women from Mexico are at greater risk of exploitation than non-migrants as a consequence of a hierarchy between migrants and non-migrants [52].

From the perspective of immigrant populations, migrants who have a partner have better chances of participating in the labor market than migrants who are single, regardless of whether the partner is another migrant or is a member of the majority group [104]. This difference in participation levels has been attributed to the spouse effect, which is the positive or the negative effect of individual resources on the labor market participation of the partner [8]. It has been argued that for mixed unions, there is an intermarriage premium effect on the labor market [72]. This concept implies that a first-generation migrant in a union with a non-migrant tends to benefit from the non-migrant partner’s social networks, which may improve the migrant’s employment chances and outcomes [34]. However, this effect seems to depend on the migration context, as it is influenced by the selectivity of the groups who intermarry [25, 60]. Whether this premium effect pays off also for non-migrants has been debated. While Meng and Gregory found no evidence of such a premium effect for non-migrants [72], Nottmeyer reported a premium for non-migrant women in a mixed union, but not for non-migrant men [82]. Based on the findings of a qualitative study, Menz [73] suggested that German women with a migrant partner from a non-European country may be more likely in a female breadwinner arrangement, as the migrant man is frequently not allowed or not able to find employment.

### Working Hypotheses

We conclude the background of our study by formulating working hypotheses to guide our analyses. Although there is an ongoing shift from male breadwinner to one-and-a-half-earner or dual-earner models, our review of gendered employment patterns and our summary of research on mixed unions lead us to expect the opposite trend for mixed unions. Based on the various aspects of labor market inequalities associated with gender and migration status, both between the migrant generations and the migrants’ regions of origin, we formulate three hypotheses about the differences in the gendered employment patterns of non-migrant/migrant mixed couples and of non-migrant endogamous unions.

First, the employment patterns in mixed unions may be the consequence of discrimination or inequalities in the labor market that affect the migrant partner. Therefore, we would expect to find that one breadwinner arrangements are more common in mixed unions, with the non-migrant partner being more likely to be employed than the migrant partner (H1a—structure hypothesis). However, as the labor market is a partner market that fosters positive assortative mating, we would—in contrast to H1a—expect to find that a mixed union is associated with a dual-earner arrangement (H1b—structure hypothesis).

Second, given the persistent gender inequalities in the labor market and in family arrangements in Germany, we assume that non-migrant men and women in mixed unions follow different gendered employment patterns. The previous literature has shown that both non-migrant women and migrant men face labor market disadvantages. Therefore, a mixed union involving a non-migrant woman and a migrant man has a lower earnings potential than a mixed union involving a non-migrant man and a migrant woman, and lower chances of realizing such a one breadwinner arrangement. Consequently, we expect to find that one breadwinner arrangements are more common among non-migrant men in mixed unions than among non-migrant women in mixed unions (H2—gender hypothesis).

Third, the employment patterns in mixed unions may be influenced by the different legal conditions that apply to European and non-European migrants. We expect to find that one breadwinner arrangements are more common for mixed unions involving a non-European partner, while intra-European couples or those involving a second-generation migrant partner differ less from endogamous unions (H3—legal conditions hypothesis).

## Data

For our analysis, we draw on the scientific use file of the German Microcensus for 2013 [21, 89]. The Microcensus is a representative sample of the German resident population, both non-migrants and migrants. A non-migrant is defined as a person who was born as a German citizen in Germany and as the child of two German citizens who were also born in Germany. Following the definition in German official statistics, we define a “migrant” as a person who was either not born in Germany and moved to Germany from abroad (a group we call the first generation), or who was born as the child of at least one parent who was born abroad and moved to Germany, or who was not born as a German citizen (a group we call the second generation) [22]. In investigating our research question regarding the gendered employment patterns of couples, we concentrate on non-migrants of working ages (18–67, neither partner is retired or in education) who are living with their partner in an opposite-sex union and in the same household. We also excluded all households in which a child under one year old or a partner on parental leave is living, as their arrangements in this time period are influenced by parenthood, and are likely to change. We use separate samples for women and men. Our analytical sample for men contains 44,499 non-migrant men (almost 7% in mixed unions) and 43,722 non-migrant women (over 5% in mixed unions). Broken down by region, 20% of the men in the sample are living in eastern Germany (8,854, of whom around 2% are in a mixed union, compared to 7% of the men in western Germany). The distribution for non-migrant women is similar.

### Variables

Our dependent variable is a nominal variable that reflects the gendered employment patterns of the couple. For the number of working hours, the following categories are distinguished for all types of gainful employment including self-employment: full-time (30 h or more), part-time (14–29 h), and marginal employment (less than 14 h). The individuals with zero working hours per week are considered unemployed or non-employed (if one of the partners is non-employed because s/he is retired or in education, we excluded the household). The categories of the variable refer to: (1) the male breadwinner model (the man is working full-time and the woman is either unemployed or non-employed); (2) the one-and-a-half-earner model (the man is working full-time and the woman is working in a part-time job or is marginally employed)^1^; (3) the dual-earner model (both the man and the woman are working either part- or full-time); (4) the female breadwinner model (the woman is working full-time and the man is unemployed or non-employed); and (5) other arrangements that we have not distinguished further (like the woman is in part-time employment and the man is unemployed, or both partners are unemployed).

Our main explanatory variable is the couple type. We distinguish three types of couples: (1) endogamous (two non-migrant partners); (2) exogamous with a European or a partner of the second migrant generation (the migrant partner has the freedom of movement associated with EU citizenship, and has been socialized in a European context); and (3) exogamous with a non-European partner.

As we want to control for potential regional differences within Germany, we use a variable to distinguish between the eastern and western German federal states. In order to control for selection into mixed unions and confounding effects, we use a number of additional socio-demographic variables. The variable educational level distinguishes between three groups: low level (primary education), medium level (secondary and post-secondary education), and high level (tertiary education). Similar information is available for the partners’ education. This enables us to account for educational homo-/heterogamy between the partners. The respondents’ and the partners’ ages are used as a categorical age variable with three seven-year groups that start with ages 18 to 33 and end with ages 58 to 67. Furthermore, we have created a variable indicating the age difference of the respondent and his/her partner. This information is pooled in three ways depending on whether the respondent is the same age, younger, or older than his/her partner. A male respondent is considered the same age as his partner if he is no more than four years older or one year younger than his partner; he is categorized as younger if he is two or more years younger than his partner; and he is considered older if he is five or more years older than his partner. For women, this categorization system is constructed in reverse. Moreover, we control for the number of children in the respondent’s household (none, one, two, 3 +) and the respondent’s marital status.

### Method

We test the gender hypothesis (H2) in a bivariate analysis. Then, we carry out multinomial logistic regression models [69], separately for men and women, in order to test the structure hypotheses (H1 a and b) and the hypothesis of legal conditions (H3). The results are displayed as average marginal effects [77]. We add in three steps independent variables starting with the main explanatory variable of the couple type (endogamous, mixed European, mixed non-European). The second models include a control for whether the respondent is living in eastern or western Germany. In order to control for potential confounders and compositional differences between the groupings, the third models include the age and the educational level of the non-migrant partner as well as of the migrant partner, measures of heterogamy in age and education between the partners, and couple-level information about the union type and the number of children in the household.

In order to check the robustness of our results, we use different strategies to account for possible regional differences, such as an interaction term for union type and region, as well as separate models for eastern and western Germany.

## Results

### Descriptive Overview by Gender and Couple Type

We show the descriptive overview for our sample of non-migrant men and non-migrant women (Table 1). The bivariate analysis reveals that the employment patterns differ for men and women in exogamous unions. In endogamous unions, the dual-earner model is the most frequent (40%), followed by the one-and-a-half-earner model (39%) and the male breadwinner model (14%). Men and women who are in a union with a European or a second-migrant generation partner are less likely to be in a dual-earner arrangement (37% for men and women). The results also indicate that compared to their counterparts in endogamous unions, non-migrants in a union with a non-European partner are slightly less likely to follow the dual-earner or the one-and-a-half-earner model. Among these unions, a one breadwinner arrangement in which the non-migrant is the main earner (32% for men and 10% for women) is more common. However, the results also show that a dual-earner arrangement is more common for women with a non-European partner than for men in such an exogamous union. Regarding our gender hypothesis (H2), we conclude that non-migrant men are more likely to be the (only) breadwinner than non-migrant women in exogamous unions.

**Table 1:** Descriptive overview of the sample in different types of union

			Endogamous				Exogamous: EU/2nd gen			Exogamous: non-European				Total			
Variables			Persons		(%)		Persons	(%)		Persons		(%)		Persons		(%)	
Descriptive overview of the sample for non-migrant men																	
Employment patterns	Male breadwinner		5,904		14.2		353	16.8		281		32.0		6,538		14.7	
	One-and-a-half-earner		16,123		38.8		836	39.7		263		30.0		17,222		38.7	
	Dual-earner		16,635		40.1		773	36.7		241		27.4		17,649		39.7	
	Female breadwinner		805		1.9		36	1.7		20		2.3		860		1.9	
	Other		2,051		4.9		106	5.0		73		8.3		2,230		5.0	
Germany	Western		32,856		79.1		1,994	94.8		795		90.5		35,645		80.1	
	Eastern		8,661		20.9		110	5.2		83		9.5		8,854		19.9	
Age	18–33		2,078		5.0		182	8.7		50		5.7		2,310		5.2	
	34–41		5,380		13.0		426	20.2		167		19.0		5,973		13.4	
	42–49		12,341		29.7		704	33.5		299		34.1		13,344		30.0	
	50–57		13,666		32.9		491	23.3		238		27.1		14,395		32.3	
	58–67		8,052		19.4		301	14.3		124		14.1		8,477		19.0	
Education	Low		13,958		33.6		658	31.3		277		31.5		14,893		33.5	
	Medium		17,223		41.5		725	34.5		289		32.9		18,237		41.0	
	High		10,336		24.9		721	34.3		312		35.5		11,369		25.5	
Educational difference to partner	Same level		25,427		61.2		1,207	57.4		478		54.4		27,112		60.9	
	Llower level		7,541		18.2		392	18.6		158		18.0		8,091		18.2	
	Higher level		8,549		20.6		505	24.0		242		27.6		9,296		20.9	
Age difference to partner	Same age		27,239		65.6		1,175	55.8		307		35.0		28,721		64.5	
	Younger		4,523		10.9		273	13.0		103		11.7		4,899		11.0	
	Older		9,755		23.5		656	31.2		468		53.3		10,879		24.4	
Marital status	Married		40,402		97.3		2,032	96.6		859		97.8		43,293		97.3	
	Non-married		1,115		2.7		72	3.4		19		2.2		1,206		2.7	
Number of children (1 year or older) in household	No children		16,520		39.8		762	36.2		352		40.1		17,634		39.6	
	1 child		11,314		27.3		566	26.9		266		30.3		12,146		27.3	
	2 children		10,697		25.8		624	29.7		214		24.4		11,535		25.9	
	3 or more children		2,986		7.2		152	7.2		46		5.2		3,184		7.2	
Age of partner	18–33		3,530		8.5		321	15.3		167		19.0		4,018		9.0	
	34–41		6,861		16.5		627	29.8		294		33.5		7,782		17.5	
	42–49		13,581		32.7		587	27.9		248		28.2		14,416		32.4	
	50–57		13,040		31.4		395	18.8		123		14.0		13,558		30.5	
	58–67		4,505		10.9		174	8.3		46		5.2		4,725		10.6	
Education of Partner	Low		12,141		29.2		676	32.1		394		44.9		13,211		29.7	
	Medium		22,349		53.8		840	39.9		163		18.6		23,352		52.5	
	High		7,027		16.9		588	27.9		321		36.6		7,936		17.8	
Total			41,517		93.3		2,104	4.7		878		2.0		44,499			
Descriptive overview of the sample for non-migrant women																	
Employment patterns	Male breadwinner	5,904		14.2		258			14.9		68		14.4		6,230		14.2
	One-and-a-half-earner	16,123		38.8		681			39.3		110		23.3		16,914		38.7
	Dual-earner	16,635		40.1		636			36.7		162		34.2		17,433		39.9
	Female breadwinner	804		1.9		42			2.4		44		9.3		890		2.0
	Other	2,051		4.9		115			6.6		89		18.8		2,255		5.2
Germany	Western	32,856		79.1		1,631			94.2		411		86.9		34,898		79.8
	Eastern	8,661		20.9		101			5.8		62		13.1		8,824		20.2
Age	18–33	3,530		8.5		300			17.3		77		16.3		3,907		8.9
	34–41	6,861		16.5		391			22.6		107		22.6		7,359		16.8
	42–49	13,581		32.7		508			29.3		136		28.8		14,225		32.5
	50–57	13,040		31.4		404			23.3		114		24.1		13,558		31.0
	58–67	4,505		10.9		129			7.4		39		8.2		4,673		10.7
Education	Low	12,141		29.2		550			31.8		161		34.0		12,852		29.4
	Medium	22,349		53.8		839			48.4		208		44.0		23,396		53.5
	High	7,027		16.9		343			19.8		104		22.0		7,474		17.1
Educational difference to partner	Same level	25,427		61.2		1,008			58.2		247		52.2		26,682		61.0
	Lower level	8,549		20.6		300			17.3		85		18.0		8,934		20.4
	Higher level	7,541		18.2		424			24.5		141		29.8		8,106		18.5
Age difference to partner	Same age	27,239		65.6		994			57.4		181		38.3		28,414		65.0
	Younger	9,755		23.5		451			26.0		128		27.1		10,334		23.6
	Older	4,523		10.9		287			16.6		164		34.7		4,974		11.4
Marital status	Married	40,402		97.3		1,678			96.9		461		97.5		42,541		97.3
	Non-married	1,115		2.7		54			3.1		12		2.5		1,181		2.7
Number of children (1 year or older) in household	No children	16,520		39.8		604			34.9		176		37.2		17,300		39.6
	1 child	11,314		27.3		478			27.6		124		26.2		11,916		27.3
	2 children	10,697		25.8		507			29.3		121		25.6		11,325		25.9
	3 or more children	2,986		7.2		143			8.3		52		11.0		3,181		7.3
Age of partner	18–33	2.078		0.0		186			10.7		64		13.5		252		0.6
	34–41	5.38		0.0		396			22.9		127		26.8		528		1.2
	42–49	12.341		0.0		496			28.6		135		28.5		643		1.5
	50–57	13.666		0.0		418			24.1		94		19.9		526		1.2
	58–67	8.052		0.0		236			13.6		53		11.2		297		0.7
Education of partner	Low	13,958		33.6		749			43.2		245		51.8		14,952		34.2
	Medium	17,223		41.5		577			33.3		100		21.1		17,900		40.9
	High	10,336		24.9		406			23.4		128		27.1		10,870		24.9
Total		41,517		95.0		1,732			4.0		473		1.1		43,722		

Source: RDC of the Federal Statistical Office and Statistical Offices of the Länder, German Microcensus SUF 2013, own calculations

Overall, we find that exogamy rates are higher among men than among women, as 5% of the men, compared with 4% of the women, are in a union with a European migrant or a descendent of a migrant. Our results further demonstrate that around 2% of the non-migrant men, compared with 1% of the non-migrant women, are in a union with a non-European migrant. The descriptive results for the employment patterns demonstrate some additional differences (Appendix Tables 4 & 5). Non-migrant men in dual-earner arrangements as well as non-migrant women who are in dual-earner or female breadwinner arrangements rather than in the other arrangement types are more likely to have medium or high educational levels, and to be living in eastern Germany. They are also more likely to have no children in the household than their counterparts in all other arrangements. Furthermore, we observe differences between non-migrant men and women in western and eastern Germany. While the shares of couples who are in male breadwinner or female breadwinner arrangements are similar in the two parts of Germany, one-and-a-half-earner arrangements are more common in western than in eastern Germany, and dual-earner arrangements are more common in eastern than in western Germany, regardless of whether one of the partners is a migrant.

### Multinomial Logistic Regression Results for Non-Migrant Men

Table 2 displays the multivariate results for non-migrant men. In the first model (Table 2A), the effect of the union type shows that non-migrants with a European/second-generation partner and non-migrants in a union with a non-European partner have a higher likelihood of being in a male breadwinner arrangement than their counterparts in an endogamous union. Accordingly, non-migrants in both types of exogamous unions are less likely to be in a dual-earner arrangement.

**Table 2:** Multinomial regression results (AME) for non-migrant men in different types of union

GFEmployment patterns of non-migrant men (*N* = 44,499)	Male breadwinner	One-and-a-half-earner	Dual-earner	Female breadwinner	Other
*2A: Model 1*					
Union type (Ref.: endogamous)					
Exogamous: Europe or 2nd generation	0.03**	0.01	− 0.03**	0.00	0.00
Exogamous: non-European	0.18***	− 0.09***	− 0.13***	0.00	0.03***
*2B: Model 2*					
Union type (Ref.: endogamous)					
Exogamous: Europe or 2nd generation	0.01	− 0.03***	0.02	0.00	0.00
Exogamous: non-European	0.16***	− 0.11***	− 0.09***	0.01	0.04***
Germany (Ref.: western)					
Eastern	− 0.07***	− 0.29***	0.32***	0.02***	0.02***
*2C: Model 3*					
Union type (Ref.: endogamous)					
Exogamous: Europe or 2nd generation		0.02**	− 0.02*	− 0.01	0.00
Exogamous: non− European		0.19***	− 0.09***	− 0.13***	0.00
Germany (Ref.: western)					
Eastern	− 0.03***	− 0.26***	0.24***	0.02***	0.04***
Age (Ref. 50–57)					
18–33	− 0.01	− 0.05**	0.06***	0.00	0.00
34–41	− 0.01	0.01	0.02	0.00	− 0.01**
42–49	− 0.01*	0.01	0.02*	− 0.01**	− 0.01*
58–67	0.02**	− 0.02	− 0.05***	0.01***	0.03***
Education (Ref.: medium)					
Low	0.00	0.00	− 0.04**	0.02*	0.03***
High	0.04***	− 0.01	− 0.01	− 0.01***	− 0.01*
Educational differences (Ref.: same level)					
Lower level	0.00	0.00	0.00	0.00	− 0.01
Higher level	0.01	0.02	− 0.05***	0.01	0.01
Age differences (Ref.: same age)					
Younger than partner	0.02**	− 0.02**	0.00	0.00	0.01*
Older than partner	− 0.01**	− 0.03***	0.01*	0.01***	0.02***
Marital type (Ref. married)					
Non− martial union	0.08***	0.13***	− 0.19***	0.00	− 0.02**
Number of children in HH (Ref.: no child)					
1	0.03***	0.19***	− 0.20***	− 0.01***	− 0.01***
2	0.07***	0.25***	− 0.29***	− 0.02***	− 0.01**
3 or more	0.17***	0.19***	− 0.35***	− 0.01***	0.00
Age of Partner (Ref. 50–57)					
18–33	0.01	− 0.05***	0.05**	0.00	
34–41	− 0.04***	0.02	0.02*	0.00	0.00
42–49	− 0.04***	0.00	0.04***	0.00	0.00
58–67	0.05***	0.00	− 0.07***	0.00	0.02***
Education of Partner (Ref.: medium)					
Low	0.09***	− 0.01	− 0.10***	− 0.01**	0.02**
High	− 0.05***	− 0.09***	0.11***	0.02*	0.01*

Source: RDC of the Federal Statistical Office and Statistical Offices of the Länder, German Microcensus SUF 2013, own calculations

*p ≤ 0.05; ** *p* ≤ 0.01; *** *p* ≤ 0.001

In Model 2 (Table 2B), we control for the differences between eastern and western Germany. The results for non-migrants in a union with a non-European partner change slightly. The results for non-migrants with a European partner change to a greater extent. The differences in the likelihood of following the male breadwinner or the dual-earner model are no longer significant, and the likelihood of following the one-and-a-half-earner model is around three percentage points lower.

In Model 3 (Table 2C), the effects of the employment patterns increase, and the differences between non-migrants in an endogamous union and non-migrants in a union with a European or second-generation migrant are again significant. Compared to their counterparts in an endogamous union, non-migrants in a union with a non-European partner are, on average, 19 percentage points more likely, and those in a union with a European/second-generation partner are two percentage points more likely, to be in a male breadwinner arrangement. The model also indicates that non-migrants in a union with a non-European partner are 13 percentage points less likely, and those in a union with a European/second-generation partner are slightly less likely, to be in a dual-earner arrangement, holding all other variables constant.

To sum up, regarding our structure hypothesis (H1a), we find that non-migrant men are more likely to be the main earner in the couple. Furthermore, regarding our legal conditions hypothesis (H3), we find more differences between endogamous unions and unions with a non-European migrant than between endogamous unions and couples with a European or second-generation migrant. However, we should acknowledge that our conclusions apply mainly to western Germany. We performed robustness checks of our results using separate models for eastern and western Germany, as well as using a model with an interaction of the region and the union type (not displayed). The results suggested that the dual-earner model was more common in mixed unions in the eastern than in the western part of the country, but that the differences in the one-and-a-half earner model by union type were only significant in western Germany. We should also note that the numbers of migrants as well as the numbers of mixed unions are rather small in the eastern German sub-sample. Therefore, we will not elaborate further on these differences here, and will instead focus on the results for the main earner. Differences by union type for this employment pattern can be found in western and eastern Germany.

### Multinomial Logistic Regression Results for Non-Migrant Women

Table 3 displays the multivariate results for non-migrant women. We find in Model 1 (Table 3A) that compared to their counterparts in endogamous unions, non-migrant women in both types of exogamous unions are as likely to be in a male breadwinner arrangement and are significantly less likely to be in a dual-earner arrangement. In addition, non-migrant women with a non-European partner are seven percentage points more likely to be in a female breadwinner arrangement.

**Table 3:** Multinomial regression results for non-migrant women in different types of union

Employment patterns of non-migrant women (*N* = 43,722)	Male breadwinner	One-and-a-half-earner	Dual− earner	Female breadwinner	Other
*3A: Model 1*					
Union type (Ref.: endogamous)					
Exogamous: Europe or 2nd generation	0.01	0.00	− 0.03**	0.00	0.02**
Exogamous: non-European	0.00	− 0.16***	− 0.06**	0.07***	0.14***
*3B: Model 2*					
Union type (Ref.: endogamous)					
Exogamous: Europe or 2nd generation	0.00	− 0.04***	0.01	0.01*	0.02**
Exogamous: non-European	− 0.01	− 0.17***	− 0.04*	0.08***	0.14***
Germany (Ref.: western)					
Eastern	− 0.07***	− 0.29***	0.32***	0.02***	0.01***
*3C: Model 3*					
Union type (Ref.: endogamous)					
Exogamous: Europe or 2nd generation	0.00	− 0.03**	0.00	0.01*	0.02***
Exogamous: non-European	− 0.01	− 0.15***	− 0.05*	0.08***	0.13***
Germany (Ref.: western)					
Eastern	− 0.03***	− 0.26***	0.24***	0.02***	0.04***
Age (Ref.: 50–57)					
18–33	0.00	− 0.05**	0.05***	− 0.01	0.01
34–41	− 0.04***	0.02	0.02*	0.00	0.00
42–49	− 0.04***	0.00	0.04***	0.00	− 0.01
58–67	0.04***	0.00	− 0.06***	0.00	0.02***
Education (Ref.: medium)					
Low	0.09***	0.01	− 0.10***	− 0.01**	0.02**
High	− 0.06***	− 0.10***	0.13***	0.02	0.02*
Educational differences (Ref.: same level)					
Lower level	0.01	0.01	− 0.04**	0.01	0.01
Higher level	− 0.01	0.02	0.00	0.00	− 0.01
Age difference (Ref.: same age)					
Younger than partner	− 0.01**	− 0.03***	0.01*	0.01***	0.02***
Older than partner	0.01*	− 0.02**	0.00	0.00	0.01*
Marital type (Ref.: married)					
Non-martial union	0.07***	0.14***	− 0.19***	0.00	− 0.03***
(Number of children in HH Ref.: no child)					
1	0.03***	0.19***	− 0.20***	− 0.01***	− 0.01***
2	0.07***	0.25***	− 0.30***	− 0.02***	− 0.01**
3 or more	0.17***	0.20***	− 0.35***	− 0.01***	0.00
Age of Partner (Ref. 50–57)					
18–33	0.01	− 0.06***	0.05**	0.00	0.00
34–41	0.00	0.01	0.01	0.00	− 0.01**
42–49	− 0.01*	0.01	0.02*	0.00	− 0.01*
58–67	0.02***	− 0.01	− 0.05***	0.01***	0.03***
Education of Partner (Ref.: medium)					
Low	0.00	− 0.02	− 0.03*	0.02*	0.03***
High	0.04***	0.00	− 0.02	− 0.01***	− 0.01**

Source: RDC of the Federal Statistical Office and Statistical Offices of the Länder, German Microcensus SUF 2013, own calculations

* *p* ≤ 0.05; ** *p* ≤ 0.01; *** *p* ≤ 0.001

In Model 2 (Table 3B), which controls for living in eastern or western Germany, the changes in the main effect found for women are similar to those observed for men. For non-migrant women, the likelihood of being in a dual-earner arrangement is no longer depending on whether the partner is a non-migrant or a European/second-generation migrant. Compared to their counterparts in endogamous unions, the likelihood of non-migrant women in both types of exogamous unions of being in other arrangements or in a female breadwinner arrangement remains significantly higher (by eight percentage points) if the migrant partner is from a non-European country.

The findings in Model 3 (Table 3C) show that the likelihood of being in a male breadwinner arrangement does not significantly differ for non-migrant women depending on whether they are in a union with a migrant or a non-migrant. Non-migrant women in a union with a non-European partner are five percentage points less likely to be in a dual-earner arrangement. Non-migrant women in both types of exogamous unions are significantly more likely to be in a female breadwinner arrangement. However, this result is only highly significant for women with a non-European partner, as women with a European partner are only one percentage point more likely to be in a female breadwinner arrangement. Again, for the same reasons as in the models for non-migrant men, we have to restrict our results for the likelihood of being in a dual-earner or a one-and-a-half-earner arrangement to western Germany.

To conclude, regarding to the structure hypothesis (H1a), we find that non-migrant women are less likely to be in a dual-earner arrangement if they have a non-European partner, and that non-migrant women in both types of exogamous unions are more likely to be responsible for the family income. Regarding the legal conditions hypothesis (H3), we find that the employment patterns differ depending on whether the migrant partner is or is not a non-European migrant.

## Conclusion and Implications

Our paper investigated how gendered employment patterns in unions are associated with labor market inequalities in societies during times of demographic, socio-cultural, political, and economic change. In Germany, female labor force participation is increasing, while gender inequalities in the labor market persist [78]. Our focus was on the potential effects of the ethnically diverse partner/marriage market on members of the non-migrant mainstream population. To the best of our knowledge, our study is the first contribution to the research on mixed unions, and not just for Germany [7, 10, 75, 76, 80], that uses quantitative representative data to focus on the non-migrant partner, and to examine their gendered employment patterns. Most previous research on this topic has focused primarily on the migrant partner in such unions who crossed social boundaries between migrant minority and majority groups by marrying a member of the majority, which may have facilitated the process of becoming part of the host society [2]. Our research question was how the employment patterns of a union are associated with exogamy. Hence, we have not focused on the assimilating function for migrants of being in a union [3] or of participating in the labor market [82]. We wanted to contribute to the research by scrutinizing classical approaches to the question of how migrants and non-migrants live together, and to the issue of how the presence of migrants contributes to social change in immigration societies [93, 94]. We explored the potential differences in the gendered employment patterns of a couple depending on whether it is a mixed migrant-non-migrant union or a non-migrant endogamous union. Thus, we examined an issue that has not been previously addressed in family research [79], which tends to focus primarily on either migrants or mainstream populations, while ignoring the potential impact of immigrants on the majority group.

The scientific use file of the German Microcensus from 2013 provided us with the opportunity to use a sample that includes a sufficient number of migrants and non-migrants to conduct a multivariate analysis. Before we discuss the implications of these findings, we wish to mention some limitations of our data. The data provided information about the gendered employment patterns in existing unions only, and not about the patterns in couples who were already separated or divorced. Mixed unions, which tend to have more (potential) power imbalances because the partners are more different than they are in endogamous unions, were shown to have a higher risk of dissolution [76]. To the extent that our focus on “surviving” unions affected our results, it may have led to an underestimation of the prevalence of one breadwinner arrangements. We have no information about the desired employment patterns for respondents who had a partner who was unemployed, and we cannot speculate here about what a respondent’s pattern would have been if his/her partner found a job. As we did not use longitudinal data, we cannot say whether the gendered employment patterns we observed were stable, or changed over the life course [107], especially given that being in a dual-earner arrangement and having no children (or no longer) in the household were found to be strongly connected. The data provided us with no information about the causes of these employment patterns: i.e., whether they resulted from a conscious/active decision by the family, a reaction to external circumstances, discrimination against the migrant partner on the labor market, or overall conditions on the regional labor market that could have affected both partners. Furthermore, there were no questions about religiosity, which is known to be a factor for different attitudes toward mixed unions [14] and gendered employment patterns. Finally, in terms of the age of our data, we wish to point out that for our analysis, we needed information on the extended migration histories of the individual’s parents as well as those of his/her partner, and on the number and the ages of the children living in the household. The Microcensus contains each of the items needed only every fourth year. Therefore, we could not use a more recent scientific use file.^2^ However, as we considered this topic starting from a theoretical point of view, and as we have no reason to believe that there have been drastic changes in the employment patterns in recent years, we think that using these data was appropriate.

Despite these limitations, our data proved useful for investigating our research question. As these data are representative for the whole resident population in Germany and the subsamples were large enough, we were able to distinguish between unions with a European and non-European migrant, and we were also able include couples living in eastern Germany, where a minority of the migrant population lives.

Our findings revealed that in contrast to the general trend in the mainstream population of a decline of the male breadwinner model [100], non-migrant men and non-migrant women who were in a mixed union – regardless of whether the partner was a European/second-generation migrant or a non-European migrant – were more likely to be participating in the labor market as the main breadwinner. Overall, we found that our structure hypothesis that one breadwinner arrangements are more common in mixed unions was supported (H1a), but that our alternative structure hypothesis that dual-earner arrangements are more common in mixed unions had to be rejected (H1b).

In order to understand the meaning of the breadwinner arrangement, we need to focus on the patterns of women and men separately. Previous studies have shown gender differences in how mixed unions are formed [71], and that the determinants of mixed unions and the meaning of a male breadwinner arrangement are different from those of a female breadwinner arrangement [64]. We found that the effects in the models were stronger for non-migrant men than for non-migrant women, especially for the unions with a non-European partner. These findings may be explained by the differences in the earnings potential of men and women and the lower family income provided by female breadwinners, or by the different conditions in which a female breadwinner model is chosen [24, 53], which led to the male breadwinner model being more common. In our bivariate analysis, we also found that our gender inequality hypothesis (H2)—i.e., that one breadwinner arrangements are more frequent for men than for women—was supported.

Our finding that the differences between endogamous unions and exogamous unions involving a non-European partner were especially large is related to our third assumption – i.e., the legal conditions hypothesis (H3) – which was partially supported by our results. Our findings demonstrated that non-migrant men and women who were in a union with a non-European migrant were more likely than non-migrants in an endogamous union or non-migrants in a union with a European/second-generation migrant to be in a male or female breadwinner arrangement. Compared to non-migrants, migrants have less host country-specific cultural capital (they have lower educational levels or are unable to have their educational levels recognized in the host country), have fewer relevant skills, and may experience skill downgrading [9]. In addition, migrants were shown to be, on average, more likely than non-migrants to have and to prefer a gendered division of work, although the extent of this preference varies by country of origin [63] and across migrant generations [56]. Attitudes and preferences were also shown to influence the employment patterns of migrant partners [61]. The negative effects of these differences between migrants and non-migrants may be greater than the positive network and employment effects of being in a union with a non-migrant partner. To conclude, our findings regarding the third hypothesis indicate that on the one hand, European couples seem to adapt to trends in the mainstream population and are not very different from endogamous couples; while on the other, couples involving a non-European partner continue to differ from non-migrant couples in terms of their labor market participation levels.

Overall, regarding the structure and gender hypotheses, we found higher shares of non-migrant men and women acting as the main breadwinner. We conclude that these patterns may increase the dependence of the migrant partner on the non-migrant partner. In such cases, the non-migrant partner then becomes responsible for the material security of the couple. This can lead to an imbalance of power and to conflicts between the partners [6]. For non-migrant men, exogamy seems to be associated with a re-traditionalization of employment patterns, as they are more likely to be in a one-earner couple. In comparison, non-migrants in endogamous unions are less likely to be in a one-earner arrangement. For women, by contrast, we found evidence of a role reversal in exogamous unions, with the woman being the main earner. This finding may indicate that these women are more likely to have precarious household conditions than non-migrant women in endogamous unions, who are more likely to be in one-and-a-half or dual-earner arrangements, which are, on average, associated with higher earnings.

Another point of interest is that a higher share of non-migrant women than of non-migrant men with a non-European partner are in a dual-earner arrangement. This may indicate that the women in such exogamous unions make up a selective group [36] of individuals who seek out and support a dual-earner arrangement, or that the mechanisms of the intermarriage premium are at play here [72].

Therefore, the meaning of this result may differ for non-migrant men and women. Modernization processes enable people to choose how and with whom they want to live. This question is particularly relevant for women, because they have more opportunities than earlier cohorts as a consequence of changing gender roles [37: p. 30]. Based on such an understanding of modernization, a non-migrant man may want to follow a male breadwinner arrangement that reinforces a gendered division of tasks. Moreover, migrants and non-migrants may differ in their gender attitudes as well as in their approaches to negotiating domestic work [59], and may have different desires about participating in the labor market. However, even if this arrangement is not the one that is preferred by the man and his migrant partner, the couple will try to achieve an economic situation and a household income that the partners can live on until the migrant partner can participate in the labor market in the desired way. This contrasts with the working and earning conditions of women and the negative effects of a female breadwinner model [64]. Therefore, this situation seems comparable to that of women in eastern Germany, who, on average, make larger contributions to the household income than women in western Germany [101]. This larger contribution is often a consequence of structural conditions and the employment situation of the male partner, but it is not necessarily associated with a higher household income [62].

In light of this study’s findings, it may be worthwhile for further research to not only look at aspects of gender norms and employment patterns [15], but to examine how both partners negotiate employment, domestic work, and family [42]. It would be interesting to investigate whether policies could foster the employment participation of migrants, and how this might change current employment patterns. Furthermore, as we found differences in employment patterns between eastern and western Germany related to different gender cultures [85], further research should investigate whether there are differences between mixed unions in eastern and western Germany due to different immigration histories in the two parts of Germany [23].

To sum up this study, the persistent disadvantage for immigrants on the labor market in Germany also affects members of the majority population who cross social boundaries in union formation. Such mixed unions contribute to social change and the rising diversity in family life for non-migrants in Germany, as well as for the migrant sub-population.

## Appendix

See Tables

**Table 4:** Descriptive overview of the sample for non-migrant men by employment patterns

		Male breadwinner	One-and-a-half-earner	Dual-earner	Female breadwinner	Other
Variables		(%)	(%)	(%)	(%)	(%)
Union Type	Endogamous	14.2	38.8	40.1	1.9	4.9
	Exogamous: Europe	16.8	39.7	36.7	1.7	5.0
	Exogamous: non-Europe	32.0	30.0	27.4	2.3	8.3
Germany	Western	16.1	44.4	33.2	1.5	4.7
	Eastern	9.0	15.7	65.5	3.6	6.2
Age	18–33	14.7	28.4	51.7	1.2	4.0
	34–41	13.7	44.0	38.3	1.1	2.9
	42–49	12.8	45.0	37.7	1.1	3.3
	50–57	14.5	37.5	41.4	2.0	4.6
	58–67	18.6	30.0	37.4	3.8	10.2
Education	Low	17.0	41.9	31.3	2.5	7.3
	Medium	12.4	35.9	45.5	2.1	4.0
	High	15.3	38.9	41.2	0.9	3.6
Educational difference to partner	Same level	13.8	36.8	42.3	1.9	5.3
	Llower level	10.2	39.4	42.6	3.1	4.8
	Higher level	21.4	43.8	29.4	1.0	4.5
Age difference to partner	Same age	14.7	39.6	39.6	1.7	4.5
	Younger	16.7	36.6	40.1	1.4	5.1
	Older	13.9	37.4	39.7	2.7	6.3
Marital status	Married	14.9	39.2	39.0	1.9	5.0
	Non-married	6.2	21.2	63.1	2.3	7.1
Number of children (1 year or older) in household	No children	13.0	23.7	53.6	2.9	6.9
	1 child	14.0	43.6	36.8	1.5	4.1
	2 children	15.3	53.6	26.9	0.8	3.3
	3 or more children	24.7	49.6	20.4	1.1	4.2
Age of Partner	18–33	15.7	30.9	47.9	1.3	4.1
	34–41	12.8	46.7	36.0	1.2	3.4
	42–49	12.4	43.6	38.9	1.6	3.6
	50–57	15.6	34.9	41.5	2.5	5.6
	58–67	21.5	28.2	35.9	3.3	11.1
Education of Partner	Low	22.7	41.8	26.0	1.7	7.8
	Medium	12.1	39.1	42.9	2.1	3.8
	High	9.1	32.3	52.7	1.9	4.0
Total	*N*	6,538	17,222	17,649	860	2,230

Source: RDC of the Federal Statistical Office and Statistical Offices of the Länder, German Microcensus SUF 2013, own calculations

4,

**Table 5:** Descriptive overview of the sample for non-migrant women by employment patterns

		Male breadwinner	One-and-a-half-earner	Dual-earner	Female breadwinner	Other
Variables		(%)	(%)	(%)	(%)	(%)
Union type	Endogamous	14.2	38.8	40.1	1.9	4.9
	Exogamous: Europe	14.9	39.3	36.7	2.4	6.6
	Exogamous: non-Europe	14.4	23.3	34.2	9.3	18.8
Germany	Western	15.6	44.5	33.3	1.6	4.9
	eastern	8.7	15.6	65.7	3.7	6.3
Age	18–33	14.7	31.0	48.3	1.5	4.6
	34–41	12.0	47.1	36.2	1.3	3.5
	42–49	11.9	43.7	39.1	1.6	3.6
	50–57	15.4	34.7	41.5	2.6	5.8
	58–67	21.1	28.2	36.2	3.3	11.1
Education	Low	22.3	42.0	26.0	1.8	8.0
	Medium	12.0	38.9	43.0	2.2	3.9
	High	7.6	32.4	53.9	1.9	4.2
Educational difference to partner	Same level	13.6	36.7	42.4	1.9	5.4
	Lower level	20.6	44.0	29.6	1.1	4.6
	Higher level	9.4	39.4	42.9	3.3	5.1
Age difference to partner	Same age	14.4	39.3	39.5	2.1	4.6
	Younger	12.9	37.7	40.2	2.8	6.5
	Older	15.8	36.3	40.3	2.0	5.6
Marital status	Married	14.5	39.2	39.2	2.0	5.1
	Non-married	5.9	20.4	63.5	2.3	7.9
Number of children (1 year or older) in household	No children	12.5	23.5	53.8	3.3	7.0
	1 child	13.7	43.5	37.0	1.6	4.3
	2 children	14.8	53.7	27.1	0.9	3.5
	3 or more children	24.2	49.7	20.4	1.1	4.6
Age of partner	18–33	14.8	27.8	51.0	1.7	4.7
	34–41	13.1	43.9	38.4	1.2	3.3
	42–49	12.0	45.2	38.1	1.3	3.4
	50–57	14.2	37.4	41.7	2.1	4.7
	58–67	18.5	30.0	37.4	3.9	10.2
Education of partner	Low	16.8	41.6	31.5	2.6	7.5
	Medium	12.0	36.0	45.8	2.1	4.1
	High	14.5	39.1	41.7	1.1	3.7
Total	*N*	6,230	16,914	17,433	890	2,255

Source: RDC of the Federal Statistical Office and Statistical Offices of the Länder, German Microcensus SUF 2013, own calculations

5.

## Footnotes

1 In our sample only 4% of the one-and-a-half-earner model would be with a woman in full-time employment and the man in a part-time job. As they may constitute a selective group, we defined them as part of the other arrangements.

2 The most recent wave (2017) is currently only available when working as a guest at the Federal Statistical Office. However, due to the COVID-19 pandemic, we are currently not allowed to travel to the Office.
